# A comparison of brain magnetic resonance imaging lesions in multiple sclerosis by race with reference to disability progression

**DOI:** 10.1186/s12974-018-1295-1

**Published:** 2018-09-05

**Authors:** Yuri Nakamura, Laura Gaetano, Takuya Matsushita, Altermatt Anna, Till Sprenger, Ernst-Wilhelm Radue, Jens Wuerfel, Lorena Bauer, Michael Amann, Koji Shinoda, Noriko Isobe, Ryo Yamasaki, Takahiko Saida, Ludwig Kappos, Jun-ichi Kira

**Affiliations:** 1 Department of Neurology, Neurological Institute, Graduate School of Medical Sciences, Kyushu University, 3-1-1 Maidashi, Higashi-ku, Fukuoka, 812-8582 Japan; 2 Medical Image Analysis Center (MIAC AG), Marktgasse 8, 4051 Basel, Switzerland; 3 Neurology and Department of Biomedicine, University Hospital Basel, Spitalstrasse 21, 4031 Basel, Switzerland; 4 Department of Biomedical Engineering, University of Basel, Marktgasse 8, 4051 Basel, Switzerland; 5 DKD Helios Klinik Wiesbaden, Aukammallee 33, 65191 Wiesbaden, Germany; 6 Biomedical Research and Education GmbH, Mittlere Strasse 91, 4031 Basel, Switzerland; 7 Klinikum rechts der Isar, Department of Neurology, Technical University of Munich, Ismaninger Str. 22, 81675 Munich, Germany; 8 Division of Diagnostic and Interventional Neuroradiology, Department of Radiology, University Hospital Basel, Spitalstrasse 21, 4031 Basel, Switzerland; 9 Department of Neurological Therapeutics, Neurological Institute, Graduate School of Medical Sciences, Kyushu University, 3-1-1 Maidashi, Higashi-ku, Fukuoka, 812-8582 Japan; 10 Institute of Neurotherapeutics, 16-1 Nishinokyoukasugachou, Nakagyo-ku, Kyoto, 604-8453 Japan; 11 Department of Neurology, Kyoto Min-Iren-Central Hospital, 16-1 Nishinokyoukasugachou, Nakagyo-ku, Kyoto, 604-8453 Japan

**Keywords:** Brain lesions, Brain volume, Disability, Magnetic resonance imaging, Multiple sclerosis, Progression, Race

## Abstract

**Background:**

We compared the magnetic resonance imaging (MRI) features between Japanese and Caucasian patients with multiple sclerosis (MS), and identified the relationships between MRI features and disability.

**Methods:**

From the baseline data of phase II fingolimod trials, 95 Japanese and 246 Caucasian relapsing-remitting MS patients were enrolled. The number, volume, and distribution of brain MRI lesions were evaluated using T2-weighted (T2W) images. Cross-sectional total normalized brain volume (NBV), normalized cortical gray matter volume, normalized deep gray matter volume (NDGMV), normalized white matter volume (NWMV), and normalized thalamic volume were measured.

**Results:**

Japanese patients had significantly lower Expanded Disability Status Scale (EDSS) scores than Caucasian patients (mean 2.0 vs. 2.3, *p* = 0.008), despite a similar disease duration. Japanese patients showed a trend towards fewer T2W-lesions (median 50 vs. 65, *p* = 0.08) and significantly lower frequencies of cerebellar and parietal lobe lesions (*p* = 0.02 for both) than Caucasian patients. There were no differences in T2W-lesion volume between races, whereas Japanese patients had a significantly larger T2W-lesion volume per lesion compared with Caucasian patients (median 140 mm^3^ vs. 85 mm^3^, *p* < 0.0001). T2W-lesion volumes were positively correlated with EDSS scores in Japanese patients (*p* < 0.0001). In both races, NBV, normalized cortical gray matter volume, NDGMV, and thalamic volume were negatively correlated with disease duration and EDSS scores (*p* < 0.01 for all). NWMV was negatively correlated with disease duration and EDSS scores only in Caucasian patients (*p* = 0.03 and *p* = 0.004, respectively). NBV, NDGMV, NWMV, and thalamic volume were consistently smaller in Japanese compared with Caucasian patients throughout the entire examined disease duration (*p* = 0.046, *p* = 0.01, *p* = 0.005, and *p* = 0.04, respectively). Japanese patients had a significantly faster reduction in NDGMV (*p* = 0.001), particularly for thalamic volume (*p* = 0.001), with disease duration compared with Caucasian patients.

**Conclusions:**

Gray matter atrophy is a common denominator for disability in Japanese and Caucasian patients. Additional contributory factors for disability include T2W-lesion volume in Japanese patients and white matter atrophy in Caucasian patients. Less frequent parietal and cerebellar involvement with fewer T2W-lesions may underlie milder disability in Japanese patients.

## Background

Multiple sclerosis (MS) is an autoimmune demyelinating disorder of the central nervous system, caused by a complex interaction between environmental and genetic factors. As such, the clinical and magnetic resonance imaging (MRI) features of MS are likely influenced by differences in genetic backgrounds between racial groups. Indeed, individuals with African ancestry, who develop MS less frequently than those with European ancestry [1], show more rapid disease progression after MS onset [2, 3].

The prevalence of MS is also low in individuals with Asian ancestry, including Japanese people. To our knowledge, only one study has conducted a direct comparison of disease severity between British and Japanese MS patients. This institutional study, which took place after the discovery of a specific biomarker for neuromyelitis optica (neuromyelitis optica IgG) revealed that Japanese MS patients exhibited milder disability compared with Caucasian patients [4]. The fourth nationwide survey of MS in Japan also showed that only 49.5% of patients with the conventional form of MS had brain lesions fulfilling the Barkhof criteria [5] after an average disease duration of 10.4 years [6]. This proportion was much lower than that for Caucasian patients, with 74% of patients with early relapsing-remitting MS (RRMS) [7], and approximately 50% of patients with clinically isolated syndrome [8], having brain lesions fulfilling the Barkhof criteria after a disease duration of 2 years.

Two studies have suggested that Japanese patients with MS may have lower cerebellar involvement and fewer intra-cortical lesions, as revealed by MRI, than Caucasian patients [9, 10]. In addition, although the Swanton modified criteria can predict the conversion from a clinically isolated syndrome to clinically definite MS according to baseline MRI findings with high sensitivity and accuracy in Western countries [11, 12], the sensitivity of these criteria was reported to be far lower in Asian individuals [13]. These observations suggest that the milder MS severity found in Japanese compared with Caucasian individuals may relate to a lower frequency of brain lesions.

To our knowledge, no studies have performed a direct MRI comparison between Asian and Caucasian MS patients using the same imaging protocol. Thus, in the present study, we directly compared brain MRI findings in Japanese and Caucasian MS patients, using pooled baseline data from the phase II fingolimod trials in Japan and Western countries, obtained using a standardized MRI protocol, with the aim to clarify the mechanisms of the milder disease course in Japanese MS patients.

## Methods

### Patients

We examined brain MRI from a population of patients with RRMS selected from pooled baseline data from phase II fingolimod trials for treatment of MS. The trials were conducted at 32 centers in 10 European countries and Canada, and at 43 centers in Japan. All participants from Europe and Canada were Caucasian, and all participants from Japan were Japanese. The eligibility criteria for the core studies were previously described in detail [14, 15]. In brief, participants were between 18 and 60 years of age, with a clinical diagnosis of relapsing MS. Patients had at least one of the following: one or more relapses in the previous year before enrollment, two or more relapses in the previous 2 years, and at least one contrast-enhanced T1-weighted (T1W) brain lesion on baseline MRI. Patients also had at least one T2-weighted (T2W) brain lesion, were neurologically stable without relapse, and had a Kurtzke’s Expanded Disability Status Scale (EDSS) score [16] ranging from 0 to 6. Exclusion criteria were use of corticosteroids (within the previous 30 days), immunomodulatory therapy (within the prior 3 months), or immunosuppressive treatment (e.g., azathioprine or methotrexate within the past 6 months).

We collected baseline demographic and clinical data including sex, age, disease duration from the initial symptom, EDSS score, Multiple Sclerosis Severity Score (MSSS), and previous MS treatment exposure such as disease-modifying drugs (DMDs) or immunosuppressive therapy. Of the participants who met the baseline eligibility criteria for the phase II fingolimod clinical trial, 167 Japanese and 250 Caucasian patients had RRMS. In the Japanese RRMS group, 70 patients did not consent to take part in the present study and were not included. Clinical parameters from two of the Japanese patients were unavailable, and these patients were excluded from this study. In the Caucasian RRMS group, four patients were excluded as their baseline scans or clinical parameters were unavailable. Demographics and MRI parameters were available for a total of 95 Japanese and 246 Caucasian patients. All participants provided written informed consent, and the research protocol for the study was approved by the Kyushu University Ethics Committee.

### Image acquisition

MRI images were obtained using 1.5 T scanners with interleaved axial 3.0-mm-thick slices, field of view = 25 cm, and matrix = 128 × 128. The imaging protocol included axial proton density (repetition time [TR] 2800–3800 ms; echo time [TE] 14–40) and T2W fast/turbo spin echo (TR 2800–3800 ms; TE 80–1200 ms). Additionally, T1W conventional spin echo (TR 500–650 ms; TE 10–20 ms) and post-contrast T1W spin-echo images (TR 500–650 ms; TE 10–20 ms) were obtained.

### MRI analysis

Trained and experienced technicians at the Medical Image Analysis Center (MIAC, Basel, Switzerland), who were blinded to clinical information, conducted T2W-lesion segmentation on T2W images using semi-automatic thresholding contour software with an interactive digital analysis program (AMIRA V.3.1.1; Mercury Computer Systems Inc., Hillsboro, OR, USA). To compare the number, volume, and distribution of T2W-lesions between the Japanese and Caucasian cohorts, we selected only patients with MRI scans that completely covered the regions from the medulla to the top of the parietal lobe. Scans from 93 Japanese and 72 Caucasian patients were available for evaluation of total T2W-lesion number and volume. We also used this dataset to calculate the average T2W-lesion volume in individuals (dividing the total T2W-lesion volume by the number of T2W-lesions) in the Japanese and Caucasian cohorts. The distribution and location of T2W-lesions were determined as follows. Segmented hyper-intense T2W-lesions in native space were linearly registered to the T1 images, which were then registered to the Montreal Neurological Institute (MNI) standard space template using a linear and nonlinear transformation method. The resulting transformation matrix was applied to the T2W-lesion mask previously registered to T1 images. We conducted a quality assessment of the procedure, and patients without successful registration to the standard were excluded from the analysis. Once the T2W-lesions were translated to MNI space, we automatically assessed the presence or absence of lesions in each brain lobe according to the Talairach atlas (i.e., frontal lobe, occipital lobe, parietal lobe, temporal lobe, limbic area, sub-lobar area, cerebellum, and brainstem), as well as the number and volume of lesions in the whole brain.

We used the entire cohort to measure total normalized brain volume (NBV), normalized cortical (NCGMV) and deep gray matter volume (NDGMV), and normalized white matter volume (NWMV) using the Structural Image Evaluation Using Normalization of Atrophy X (SIENAX) program [17]. This software uses a fully automated algorithm to estimate cross-sectional brain volume using a single time-point scan [18], performs segmentation of the brain from non-brain tissue in the head, and estimates the outer skull surface. The brain and skull images are then registered to a standard space brain and skull image derived from the MNI152 standard image. This step enables estimation of the volumetric scaling factor used for normalization of head size. To increase the reproducibility among patients, NBV, NWMV, NCGMV, and NDGMV were assessed in the 70-mm central area of the brain (z-block; MNI152 z-coordinates − 10-mm bottom to + 60-mm top). Finally, we separately assessed the normalized thalamic volume from the NDGMV using the FMRIB’s Integrated Registration and Segmentation Tool algorithm [19]. We performed a quality assessment to ensure the correct segmentation of each compartment.

T2W-lesions of 68 Japanese and 57 Caucasian patients were successfully registered to the MNI space and included in the final analysis. When comparing clinical features between the Japanese and Caucasian subgroups for evaluation of T2W-lesion distribution, we found similar trends in the demographic features to those in the total study population (data not shown). Based on white matter, cortical, and deep gray matter segmentation, scans from 83 Japanese and 233 Caucasian patients were available for NBV comparison, from 45 Japanese and 148 Caucasian patients for evaluation of NWMV, from 52 Japanese and 165 Caucasian patients for evaluation of NCGMV, and from 73 Japanese and 207 Caucasian patients for evaluation of NDGMV and normalized thalamic volume. When comparing clinical features in the Japanese and Caucasian subgroups of brain volume measurement, we found similar trends to the demographic features (data not shown).

### Statistical analyses

All analyses were performed using statistical software (JMP 13.0.0; SAS Institute Inc., Cary, NC, USA) or R. The Fisher’s exact probability test was used to compare categorical variables, while the Mann–Whitney U test was used to compare continuous variables, including age, age at onset, disease duration from the first symptom, EDSS score, MSSS, and MRI parameters, between Japanese and Caucasian patients. Race-based differences in MRI parameters, number and volumes of T2W-lesions, individual T2W-lesion size, NBV, NWMV, NCGMV, NDGMV, and normalized thalamic volume were assessed by multivariate linear regression analyses adjusting for age, sex, disease duration from the initial symptom, and MS treatment exposure. To meet the normality assumption for the regression model, the cube root transformation was applied to the number or volume of the total T2W-lesions, or individual T2W-lesion size prior to data analysis. Correlations of EDSS scores with brain volumes were examined using Spearman’s correlation. Linear regression analysis was used to examine the association of disease duration with brain volume. We also performed a test of homogeneity of slope to examine differences in the rate of brain volume reduction with disease duration, and a test of homogeneity of intercept to examine differences in brain volume at the early course of the disease, between the two racial groups. Statistical significance was set at *p* < 0.05. A statistical trend was assumed if *p* < 0.1.

## Results

### Clinical characteristics

The demographics and clinical characteristics of the Japanese and Caucasian groups are shown in Table 1 (see “Entire study population”). The proportion of women and duration of disease from the first symptom were similar between the Japanese and Caucasian groups. In the total study population, Japanese patients were younger at the time of MRI scans (mean 34.2 vs. 36.9 years, respectively; *p* = 0.03) and at the onset of the first symptom (mean 26.9 vs. 29.0 years, respectively; *p* = 0.03), and had lower EDSS scores (mean 2.0 vs. 2.3, respectively; *p* = 0.008) and MSSS (mean 3.24 vs. 3.84, respectively; *p* = 0.01), compared with Caucasian patients. The proportion of patients with previous MS treatment exposure, including DMDs or immunosuppressive therapy, was significantly higher in Japanese patients compared with Caucasian patients (56.8% vs. 28.5%, respectively; *p* < 0.0001). The demographics of the subset used for the T2W-lesion evaluation are summarized in Table 1 (see “Population for T2W-lesion evaluation”). Japanese patients in this subset also had significantly lower MSSS (mean 3.19 vs. 3.78, respectively; *p* = 0.04) and a higher frequency of previous MS treatment exposure (57.0% vs. 30.6%, respectively; *p* = 0.0009) compared with Caucasian patients.

**Table 1:** Comparison of baseline characteristics between Japanese and Caucasian patients with MS

	Entire study population			Population for T2W-lesion evaluation		
	Japanese patients (*n* = 95)	Caucasian patients (*n* = 246)	*p*	Japanese patients (*n* = 93)	Caucasian patients (*n* = 72)	*p*
Female (%)	65 (68.4)	179 (72.8)	0.43	65 (69.9)	48 (66.7)	0.74
Age at time of the MRI, years^a^	34.2 ± 8.3	36.9 ± 9.5	0.03	34.0 ± 8.3	35.9 ± 9.8	0.24
Age at first symptom, years^a^	26.9 ± 8.9	29.0 ± 8.5	0.03	26.6 ± 8.7	28.3 ± 8.6	0.15
Duration of disease from first symptom, years^a^	7.4 ± 6.4	7.9 ± 7.2	0.60	7.4 ± 6.4	7.7 ± 8.1	0.75
EDSS scores^a^	2.0 ± 1.7	2.3 ± 1.2	0.008	1.9 ± 1.7	2.2 ± 1.3	0.10
MSSS^a^	3.24 ± 2.61	3.84 ± 2.09	0.01	3.19 ± 2.60	3.78 ± 1.92	0.04
Previous exposure of DMDs or immunosuppressive therapy, *n* (%)	54 (56.8)	70 (28.5)	< 0.0001	53 (57.0)	22 (30.6)	0.0009
Patients with contrast-enhanced lesions, *n* (%)	53 (55.8)	126 (51.2)	0.47	52 (55.9)	40 (55.6)	1.00

^a^Values are expressed as mean ± standard deviation

*DMDs* Disease modifying drugs, *EDSS* Kurtzke’s Expanded Disability Status Scale, *MS* multiple sclerosis, *MSSS* Multiple Sclerosis Severity Score, *T2W* T2-weighted

### Comparison of MRI findings between Japanese and Caucasian patients with MS

**Fig. 1:**
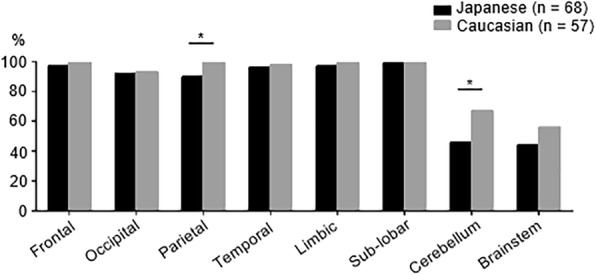
Distribution of lesions between Japanese and Caucasian patients with multiple sclerosis (MS). The proportion of patients with lesions in each brain region is shown. The proportion of patients with lesions in the cerebellum and parietal lobe was significantly lower in the Japanese group (black bar) compared with the Caucasian group (gray bar). “Sub-lobar” includes the basal ganglia and surrounding white matter. *p < 0.05

After adjusting for age, sex, duration of disease from the first symptom, and previous MS treatment exposure, Japanese patients showed a trend towards fewer T2W-lesions than Caucasian patients (median 50 vs. 65, respectively; *p* = 0.08; Table 2). However, there were no significant differences in the volume of T2W-lesions between the two groups (median 7.8 cm^3^ vs. 5.3 cm^3^, respectively; *p* = 0.12). Japanese patients had a significantly larger mean volume per lesion compared with Caucasian patients (median 140 mm^3^ vs. 85 mm^3^, respectively; *p* < 0.0001). The proportion of patients with cerebellar lesions was significantly lower in Japanese patients compared with Caucasian patients (45.6% vs. 66.7%, respectively; *p* = 0.02; Fig. 1), which was also observed for patients with lesions in the parietal lobe (89.7% vs. 100%, respectively; *p* = 0.02). There were no differences in any other brain regions between the two groups.

**Table 2:** Comparison of baseline brain MRI features between Japanese and Caucasian patients with MS

	Mean	Median (IQR)	Mean	Median (IQR)	*p*
	Japanese patients (*n* = 93)		Caucasian patients (*n* = 72)		
Number of T2W-lesions	62	50 (27–96)	73	65 (24–108)	0.08
T2W-lesion volumes (cm^3^)	11.5	7.8 (2.3–16.9)	8.0	5.3 (2.4–12.2)	0.12
Each T2W-lesion volume (mm^3^)	174	140 (78–224)	118	85 (63–138)	< 0.0001
	Japanese patients (*n* = 83)		Caucasian patients (*n* = 233)		
NBV (cm^3^)	942.6	954.1 (903.9–980.2)	972.9	977.9 (940.4–1002.6)	< 0.0001
	Japanese patients (*n* = 45)		Caucasian patients (n = 148)		
NWMV (cm^3^)	467.4	466.1 (446.5–486.1)	486.9	487.7 (473.2–501.4)	< 0.0001
	Japanese patients (*n* = 52)		Caucasian patients (*n* = 165)		
NCGMV (cm^3^)	441.3	438.3 (418.6–473.1)	449.8	452.0 (429.9–471.7)	0.08
	Japanese patients (*n* = 73)		Caucasian patients (*n* = 207)		
NDGMV (cm^3^)	37.0	36.7 (33.3–40.6)	40.8	41.4 (38.0–43.8)	< 0.0001
Normalized thalamic volume (cm^3^)	17.2	17.3 (15.7–19.0)	18.8	18.9 (17.6–20.2)	< 0.0001

Statistical comparisons were performed using linear regression with adjustments for age, sex, duration of disease from the first symptom, and exposure to previous MS treatment. We calculated the average T2W-lesion volume by dividing overall T2W-lesion volume by the number of T2W-lesions in individual patients

*IQR* interquartile range, *MRI* Magnetic resonance imaging, *MS* multiple sclerosis, *NBV* normalized brain volume, *NCGMV* normalized cortical gray matter volume, *NDGMV* normalized deep gray matter volume, *NWMV* normalized white matter volume

After adjusting for confounding factors, Japanese patients had significantly smaller NBV, NWMV, and NDGMV compared with Caucasian patients (NBV median 954.1 cm^3^ vs. 977.9 cm^3^, respectively; *p* < 0.0001; NWMV median 466.1 cm^3^ vs. 487.7 cm^3^, respectively, *p* < 0.0001; NDGMV median 36.7 cm^3^ vs. 41.4 cm^3^, respectively; *p* < 0.0001; Table 2). Among NDGMV, normalized thalamic volume of Japanese patients was also significantly smaller than that of Caucasian patients (median 17.3 cm^3^ vs. 18.9 cm^3^, respectively; *p* < 0.0001). Japanese patients also showed a trend towards a smaller NCGMV compared with Caucasian patients (median 438.3 cm^3^ vs. 452.0 cm^3^, respectively; *p* = 0.08).

### Correlation of brain MRI metrics with EDSS scores in Japanese and Caucasian MS patients

**Fig. 2:**
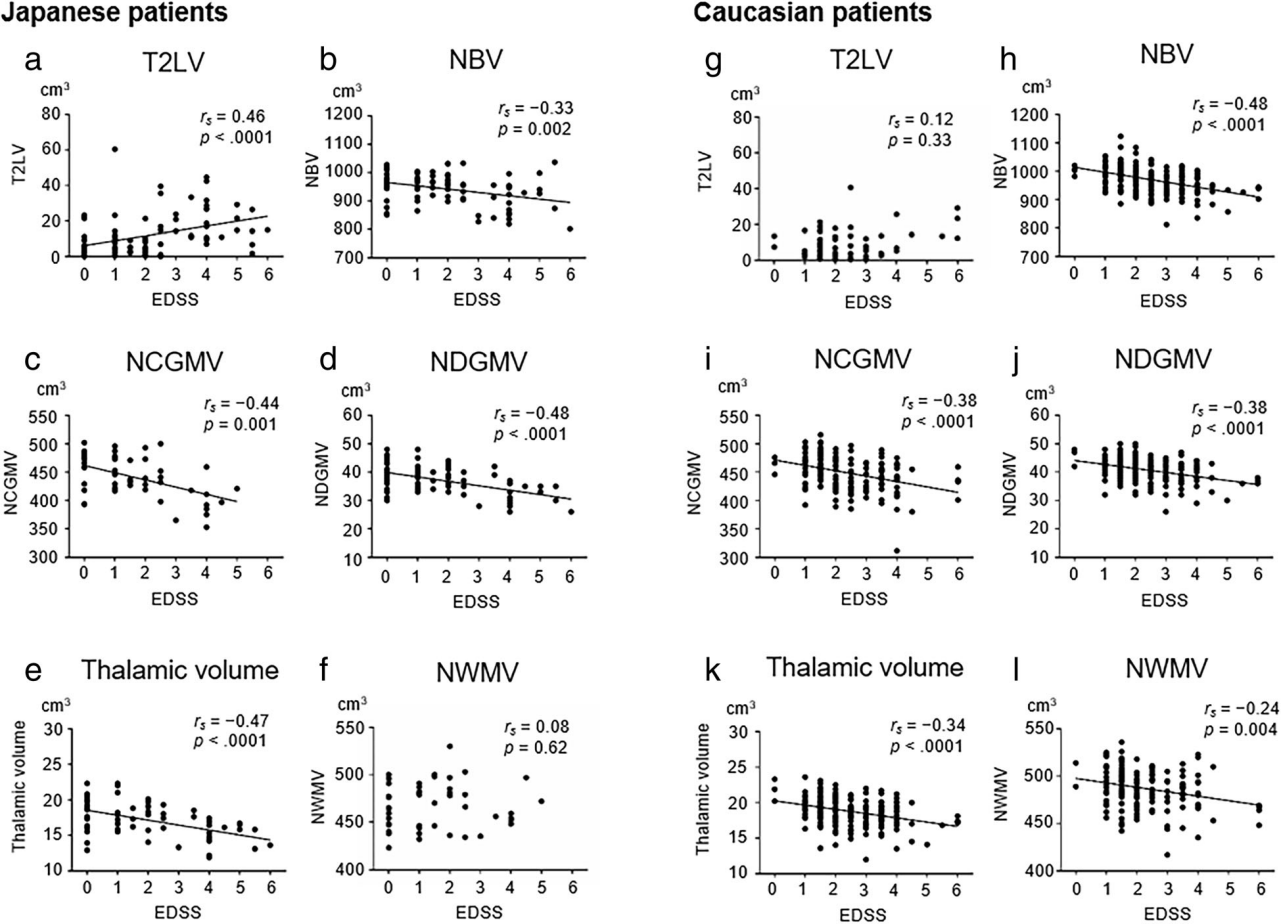
Correlations of Expanded Disability Status Scale (EDSS) scores with brain lesions or volume in patients with MS. Correlations of EDSS scores with T2W-lesion volumes (T2LV), normalized brain volume (NBV), normalized white matter volume (NWMV), normalized cortical gray matter volume (NCGMV), normalized thalamic volume, or normalized deep gray matter volume (NDGMV) in Japanese (**a–f**) and Caucasian patients (**g–l**). The lines represent the linear regression of the data. *r*_*s*_ = Spearman’s rank correlation coefficient

EDSS scores were correlated with T2W-lesion volumes in Japanese patients (*r*_*s*_ = 0.46, *p* < 0.0001; Fig. 2a), but not Caucasian patients (*r*_*s*_ = 0.12, *p* = 0.33; Fig. 2g). NBV, NCGMV, and NDGMV were significantly correlated with EDSS scores in both Japanese patients (NBV *r*_*s*_ = − 0.33, *p* = 0.002; NCGMV *r*_*s*_ = − 0.44, *p* = 0.001; NDGMV *r*_*s*_ = − 0.48, *p* < 0.0001; Fig. 2b–d) and Caucasian patients (NBV *r*_*s*_ = − 0.48, *p* < 0.0001; NCGMV *r*_*s*_ = − 0.38, *p* < 0.0001; NDGMV *r*_*s*_ = − 0.38, *p* < 0.0001; Fig. 2h–j). In both populations, EDSS scores were also negatively correlated with normalized thalamic volume (Japanese *r*_*s*_ = − 0.47, *p* < 0.0001; Caucasian *r*_*s*_ = − 0.34, *p* < 0.0001; Fig. 2e and k). There was a weak correlation of NWMV with EDSS scores in Caucasian patients (*r*_*s*_ = − 0.24, *p* = 0.004; Fig. 2l), but not in Japanese patients (*r*_*s*_ = 0.08, *p* = 0.62; Fig. 2f).

### Association of brain volume with disease duration in MS patients

**Fig. 3:**
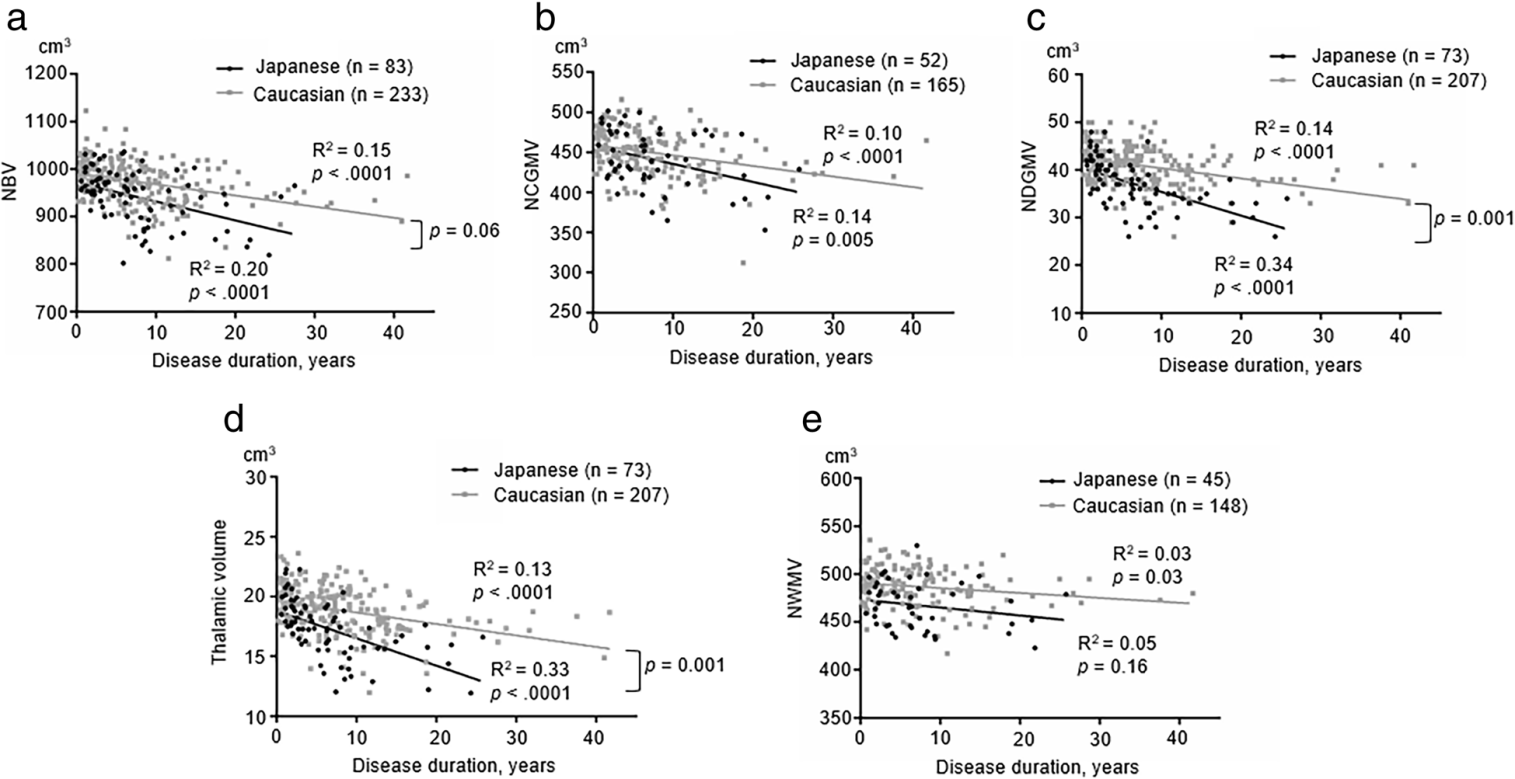
Relationship between disease duration and brain volumes in MS. Scatter plots show the relationship between disease duration and brain volumes (NBV, NCGMV, NDGMV, normalized thalamic volume, or NWMV; **a–e**) in Japanese (black dots) and Caucasian patients (gray dots). The solid lines represent the linear regression of the data. A test of homogeneity of slopes shows significant differences in NDGMV and normalized thalamic volume between the groups (*p* = 0.001 for both), and a statistically different trend in NBV between the groups (*p* = 0.06). A test of homogeneity of intercept demonstrated that NBV, NWMV, NDGMV, and normalized thalamic volume were consistently smaller in Japanese versus Caucasian patients throughout the entire disease duration examined (*p* = 0.046, *p* = 0.005, *p* = 0.01, and *p* = 0.04, respectively), while NCGMV was similar between the two racial groups in the early course of the disease. *R*^2^ = coefficient of determination

To assess the trajectory of brain volume reduction with respect to disease duration from the first symptom, we analyzed the association of disease duration with brain volume. NBV (Japanese *R*^2^ = 0.20, *p* < 0.0001; Caucasian *R*^2^ = 0.15, *p* < 0.0001), NCGMV (Japanese *R*^2^ = 0.14, *p* = 0.005; Caucasian *R*^2^ = 0.10, *p* < 0.0001), and NDGMV (Japanese *R*^2^ = 0.34, *p* < 0.0001; Caucasian *R*^2^ = 0.14, *p* < 0.0001) were negatively associated with disease duration in both cohorts (Fig. 3a–c). Normalized thalamic volume was also negatively associated with disease duration in both cohorts (Japanese *R*^2^ = 0.33, *p* < 0.0001; Caucasian *R*^2^ = 0.13, *p* < 0.0001; Fig. 3d). By contrast, NWMV was weakly and negatively correlated with disease duration in Caucasian patients (*R*^2^ = 0.03, *p* = 0.03; Fig. 3e), but not Japanese patients (*R*^2^ = 0.05, *p* = 0.16).

Finally, we used a test for homogeneity of slope to compare trends in the association of brain volume with disease duration among the Japanese and Caucasian patients. Japanese patients had a significantly faster reduction in NDGMV including normalized thalamic volume and normalized thalamic volume alone with disease duration when compared with Caucasian patients (*p* = 0.001 for both). Japanese patients also had a marginally faster reduction in NBV with disease duration compared with Caucasian patients (*p* = 0.06). These findings suggest the potential for faster deep gray matter atrophy in Japanese MS patients. NBV, NDGMV, NWMV, and normalized thalamic volume were consistently smaller in Japanese patients compared with Caucasian patients throughout the entire examined disease duration (*p* = 0.046, *p* = 0.01, *p* = 0.005, and *p* = 0.04, respectively), suggesting an inherently smaller volume of deep gray matter, white matter, and the whole brain in Japanese MS patients. NCGMV was similar between the two racial groups in the early course of the disease, and did not differ significantly in terms of the reduction slope (*p* = 0.87 and *p* = 0.22, respectively).

**Fig. 4:**
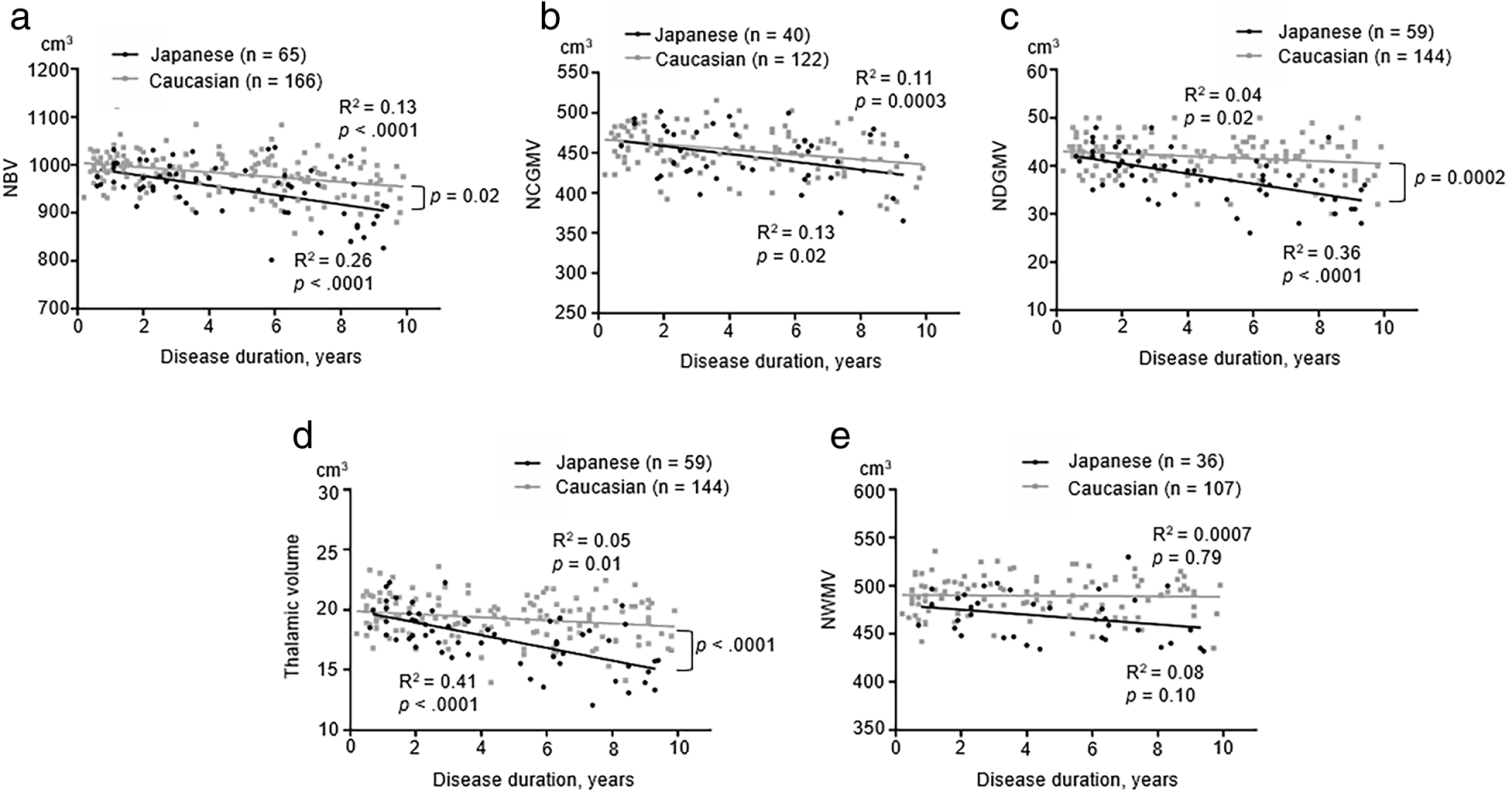
Relationship between disease duration and brain volumes in MS with a disease duration of < 10 years. Scatter plots show the relationship between disease duration and brain volumes (NBV, NCGMV, NDGMV, normalized thalamic volume, or NWMV; **a**–**e**) in Japanese (black dots) and Caucasian patients (gray dots). The solid lines represent the linear regression of the data. A test of homogeneity of slopes showed significant differences in NBV (*p* = 0.02), NDGMV (*p* = 0.0002), and normalized thalamic volume (*p* < 0.0001) between the two groups. *R*^2^ = coefficient of determination

To exclude the impact of outliers who had a disease duration of ≥ 10 years, we also evaluated the association of disease duration with brain volumes in MS patients with a disease duration of < 10 years. Even after excluding patients with a disease duration of ≥ 10 years, NBV (Japanese *R*^2^ = 0.26, *p* < 0.0001; Caucasian *R*^2^ = 0.13, *p* < 0.0001), NCGMV (Japanese *R*^2^ = 0.13, *p* = 0.02; Caucasian *R*^2^ = 0.11, *p* = 0.0003), NDGMV (Japanese *R*^2^ = 0.36, *p* < 0.0001; Caucasian *R*^2^ = 0.04, *p* = 0.02), and normalized thalamic volume (Japanese *R*^2^ = 0.41, *p* < 0.0001; Caucasian *R*^2^ = 0.05, *p* = 0.01) were negatively associated with disease duration in both cohorts (Fig. 4a–d). By contrast, NWMV was not associated with disease duration in either cohort (Fig. 4e). A test of homogeneity of slopes showed significant differences in NBV (*p* = 0.02), NDGMV (*p* = 0.0002), and normalized thalamic volume (*p* < 0.0001) between the two groups. Thus, the exclusion of outliners with a disease duration of ≥ 10 years showed a stronger trend towards Japanese patients having a faster reduction in NBV, NDGMV, and normalized thalamic volume with disease duration than Caucasian patients.

## Discussion

This study represents the first direct comparison of brain MRI findings between Japanese and Caucasian MS patients with similar disease duration using a standardized imaging protocol. The similarities in brain MRI findings between Japanese and Caucasian patients were as follows: (1) both Japanese and Caucasian patients had similar total T2W-lesion volumes, and (2) NBV, NCGMV, NDGMV, and normalized thalamic volume were negatively correlated with disease duration and EDSS scores in both racial groups. The differences in brain MRI findings were as follows: (1) Japanese patients had a larger individual lesion size compared with Caucasian patients, although Japanese patients tended to have fewer T2W-lesions. (2) Cerebellar and parietal lobe lesions were less common in Japanese patients compared with Caucasian patients. (3) There was a positive correlation of T2W-lesion volumes with EDSS scores in Japanese patients, but not in Caucasian patients. (4) NBV, NWMV, NDGMV, and normalized thalamic volume were significantly and consistently smaller in the Japanese cohort compared with Caucasian patients, while there were no differences in NCGMV. (5) The reduction in NDGMV, including normalized thalamic volume and normalized thalamic volume alone with disease duration, was faster in Japanese versus Caucasian patients. (6) NWMV was significantly and negatively correlated with EDSS and disease duration in Caucasian patients, but not in Japanese patients.

The Japanese and Caucasian MS patients in our study cohort consisted mainly of patients in the relatively early stage of RRMS, as those with progressive MS and severe disability were excluded. Although the disease duration was similar, both EDSS scores and MSSS were significantly lower in Japanese versus Caucasian patients, consistent with a previous report [4]. The average age at onset and MRI examination was slightly lower in Japanese versus Caucasian patients, although the effects of onset age on disability progression speed remain controversial [20, 21]. Indeed, it is unlikely that such a subtle difference in age had a significant impact on differences in disability between the two racial groups. Prior use of DMDs and immunosuppressants was significantly higher in Japanese versus Caucasian patients in our study (56.8% vs. 28.5%, respectively) and in the aforementioned study (83.2% vs. 17.0%, respectively) [4]. Given that DMDs and immunosuppressants have suppressive effects on relapse and new lesion formation, especially in early RRMS patients, more frequent use of these drugs may explain the lower level of disability observed in Japanese patients. Given this consideration, we carefully interpreted our MRI findings in relation to disability.

In MS, T2W-lesions are attributable to a variety of pathological reactions, including inflammatory demyelination, gliosis, edema, and axonal swelling and loss [22, 23], whereas brain volume loss reflects neuro-axonal degeneration [24]. Both processes contribute to disability progression. Inflammation was proposed to occur in the early course of MS, while neuro-axonal degeneration plays a critical role, and is more closely related to disability, in the later progressive stage [25, 26]. Nevertheless, a recent study provided evidence of early and persisting neuro-axonal degeneration throughout MS [27]. In the present study, although T2W-lesion volumes were similar between the Japanese and Caucasian patients, there was a trend towards a reduction in total T2W-lesion numbers in Japanese patients, and significantly lower frequencies of cerebellar and parietal lobe lesions. Fewer lesions in these structures may partly account for the lower degree of disability observed in Japanese patients. In particular, early cerebellar involvement is a predictor for disability progression in MS, given the abundant connections between the cerebellum and many cerebral cortical areas [28]. Additionally, the parietal lobe was reported to be associated with both physical disability and cognitive decline in MS [29, 30].

Genetic background may also influence the number of brain lesions in MS patients. We previously reported that MS patients with HLA-DRB1*04:05, one of the strongest and most common susceptibility alleles for MS in Japanese individuals, but which is rare in general European populations, showed decreased Barkhof criteria fulfillment and fewer intracortical lesions compared with those without DRB1*04:05 [10, 31, 32]. Thus, the relatively high proportion of DRB1*04:05 carriers in Japanese MS patients (approximately 30–40% in our previous report [31]) may explain the lower number of brain MRI lesions and lower degree of disability in this population. However, as described, the wide use of DMDs during the early course of disease in Japanese MS patients may also influence disability and lesion formation.

Surprisingly, Japanese patients had larger individual lesion sizes compared with Caucasian patients. In Asian MS patients, tumefactive MS may be more frequent, with an occurrence of 6.3% versus 0.1–1.4% in Western MS patients [33–35]. The development of tumefactive demyelinating lesions is attributed to the failure of focal immune-regulation against inflammatory insults that underlie MS lesion formation [36]. Although the molecular mechanisms that produce larger demyelinating lesions are unclear, demyelinating lesions may tend to expand in Japanese patients once developed. Indeed, the significant positive correlation of T2W-lesion volumes with EDSS scores in Japanese patients suggests that acute inflammation leading to T2W-lesion formation may contribute to disability in this population, and that early introduction of DMDs may be of particular benefit. Interestingly, T2W-lesion volumes, but not NWMV, correlated with EDSS scores in Japanese patients. Conversely, NWMV, but not T2W-lesion volumes, correlated with disability in Caucasian patients. This discrepancy may be based on the idea that white matter atrophy reflects a degenerative process, but is partly independent of the genesis of T2W-lesions produced by acute inflammation [24, 37]. Acute inflammation leading to T2W-lesion formation is important in Japanese patients, while white matter degeneration may contribute to disability in Caucasian patients.

In patients from Western countries, brain atrophy is significantly correlated with overall disability as evaluated by EDSS and the Multiple Sclerosis Functional Composite scores [38, 39]. In particular, gray matter atrophy was the most significant MRI variable influencing disability in Western MS patients [40]. In agreement with these observations, our study demonstrated that NBV was significantly correlated with EDSS scores, and that the correlations of NCGMV with EDSS scores, and of NDGMV with EDSS scores, were more significant than that of NWMV with EDSS scores. Additionally, normalized thalamic volume was significantly correlated with EDSS scores in both cohorts. Azevedo et al. reported that thalamic atrophy is an excellent marker for neurodegeneration in MS [27]. Therefore, gray matter atrophy including thalamic atrophy appears to be critical for determining disability between racial groups, and NCGMV, NDGMV, and normalized thalamic volume may be excellent biomarkers for disease severity across race.

Unexpectedly, we found that Japanese patients had a lower NBV, NWMV, NDGMV, and normalized thalamic volume than Caucasian patients throughout the entire disease duration, despite milder disability. To our knowledge, there are no studies that directly compare brain volume, including deep gray matter volume, between Caucasians and Japanese. However, Japanese MS patients may have an inherently smaller brain size, including the deep gray matter and white matter, after applying the normalization procedures. As white matter volume accounts for the largest proportion of whole brain volume, the initial or inherent difference in NWMV may largely explain the difference in NBV between the two groups. By contrast, the faster reduction in NDGMV and the normalized thalamic volume observed in Japanese versus Caucasian patients suggests that deep gray matter, especially the thalamus, may be lost earlier in the disease process in Japanese patients. In a comparative study of African American and Caucasian patients with MS, thalamic volume was significantly smaller in Caucasian patients, although they had lower EDSS scores than African American patients [41]. Thus, while the speed of deep gray matter loss, especially thalamic volume loss, may vary between the racial groups, its influence on physical disability may be limited, at least in the early course of the disease. However, the influence of deep gray matter degradation on cognitive function should be carefully evaluated in long-term follow-up studies.

Our study has several limitations. First, as we compared T2W-lesions between racial subgroups extracted from the entire study population, the sample sizes became relatively small. However, as the clinical characteristics between the subgroups were essentially the same as those in the entire study population, we consider the selection bias to be minimal. Although 72 of the 167 Japanese participants in the phase II fingolimod clinical trial were excluded because of lack of informed consent and data unavailability, the clinical characteristics of 95 patients enrolled in the present study were similar to that reported for the whole cohort [15]. Thus, we consider that this selection bias did not severely distort our data. Second, we could not compare the imaging measures with age-matched healthy controls in each population, as the phase II fingolimod clinical trials did not include healthy controls. Given that brain atrophy is related to normal aging processes [42] and the influence of genetic background on brain structure [43], a comparison with healthy controls is necessary to adjust for racial background in future studies. Third, we applied the MNI152 standard template, based on data from 152 individuals from Western countries, to normalize the measures for head size in both racial groups. Although this template is the most widely used in research studies and clinical trials, bias and error may occur when applying it to Japanese populations. Finally, spinal cord data were not available in this study, as the phase II fingolimod clinical trials were not designed to include spinal cord MRI. As physical disability in MS also relates to spinal cord atrophy [44], we are now prospectively measuring spinal cord cross-sectional areas and whole brain parenchymal volume in our cohort. This study will provide more information on the relationship of disability progression with neuroimaging findings.

## Conclusions

In conclusion, the cerebellum and parietal lobe are less frequently involved in Japanese MS patients compared with Caucasian patients, which may relate to milder levels of disability. Larger individual lesion sizes and a positive correlation of T2W-lesion volumes with EDSS scores in Japanese MS patients may relate to the greater role of inflammation in determining disability in Japanese than in Caucasian patients. However, brain volumes, especially gray matter volumes, are negatively associated with disease duration and disability in both Japanese and Caucasian patients, suggesting that gray matter atrophy is a common denominator for disability, and that it may be useful for evaluating disease progression. Finally, the possibility that Japanese MS patients have faster deep gray matter volume loss over time compared with Caucasian MS patients warrants future longitudinal studies.

## Abbreviations

DMDs: Disease-modifying drugs
EDSS: Expanded Disability Status Scale
MNI: Montreal Neurological Institute
MRI: Magnetic resonance imaging
MS: Multiple sclerosis
MSSS: Multiple Sclerosis Severity Score
NBV: Normalized brain volume
NCGMV: Normalized cortical gray matter volume
NDGMV: Normalized deep gray matter volume
NMO: Neuromyelitis optica
NWMV: Normalized white matter volume
RRMS: Relapsing-remitting MS
T1W: T1-weighted
T2W: T2-weighted
